# Antimicrobial and Antibiofilm Activity of an Endophytic Fungus, *Daldinia* sp. Isolated From *Avicennia officinalis*

**DOI:** 10.1002/mbo3.70422

**Published:** 2026-09-29

**Authors:** Nickita Dutta, Nutan Kumari, Anushka Lather, Kundan Kumar

**Affiliations:** ^1^ Department of Biological Sciences Birla Institute of Technology & Science Pilani, K. K. Birla Goa Campus Goa India

**Keywords:** antibacterial activity, antibiofilm activity, bioactive metabolites, endophytic fungi, mangrove

## Abstract

Mangrove ecosystems are dynamic coastal habitats that support distinctive microbial communities capable of producing structurally diverse and biologically potent secondary metabolites. Among these, endophytic fungi residing asymptomatically within mangrove plants represent an underexplored resource with substantial potential for the discovery of novel antimicrobial agents. In this study, an endophytic fungus (*Daldinia* sp.) was isolated from the leaves of *Avicennia officinalis* collected from the mangrove forests of Goa. The ethyl acetate extract of the identified endophyte demonstrated significant antibacterial activity, with zones of inhibition ranging from 13.67 ± 0.57 to 17.33 ± 0.57 mm and minimum inhibitory concentrations ranging from 312.5 to 625 μg/mL against *Escherichia coli* and *Shigella* sp., following Clinical and Laboratory Standards Institute guidelines. Flow cytometry analysis of the extract showed 84.04% ± 5.89% and 62.15% ± 1.77% of cells stained with propidium iodide (PI) in *E. coli* and *Shigella* sp., respectively, indicating substantial membrane damage. The extract induced the generation of reactive oxygen species in *E. coli* and *Shigella* sp., leading to an increase in 2′,7′‐dichlorofluorescein (DCF) fluorescence intensity. Furthermore, the extract exhibited antibiofilm activity, inhibiting biofilm formation by 93.03% ± 1.67% in *E. coli* and 89.38% ± 0.91% in *Shigella* sp. Scanning electron microscopy also supports the antibiofilm and antibacterial potential of the extract, as evidenced by reduced biofilm density and visible physical damage to bacterial cells, respectively. The metabolite profiling indicates the presence of various metabolites with antimicrobial properties. Collectively, the findings underscore the potential of an isolated endophytic fungus as a valuable source of potent antibacterial and antibiofilm compounds.

## Graphical abstract

An endophytic fungus (*Daldinia* sp.) was isolated from the leaves of *Avicennia officinalis* collected from mangrove forests of Goa. The ethyl acetate extract of *Avicennia officinalis* (AO‐EA) of the identified endophyte demonstrated antibacterial and antibiofilm activity against *Escherichia coli* and *Shigella* sp.

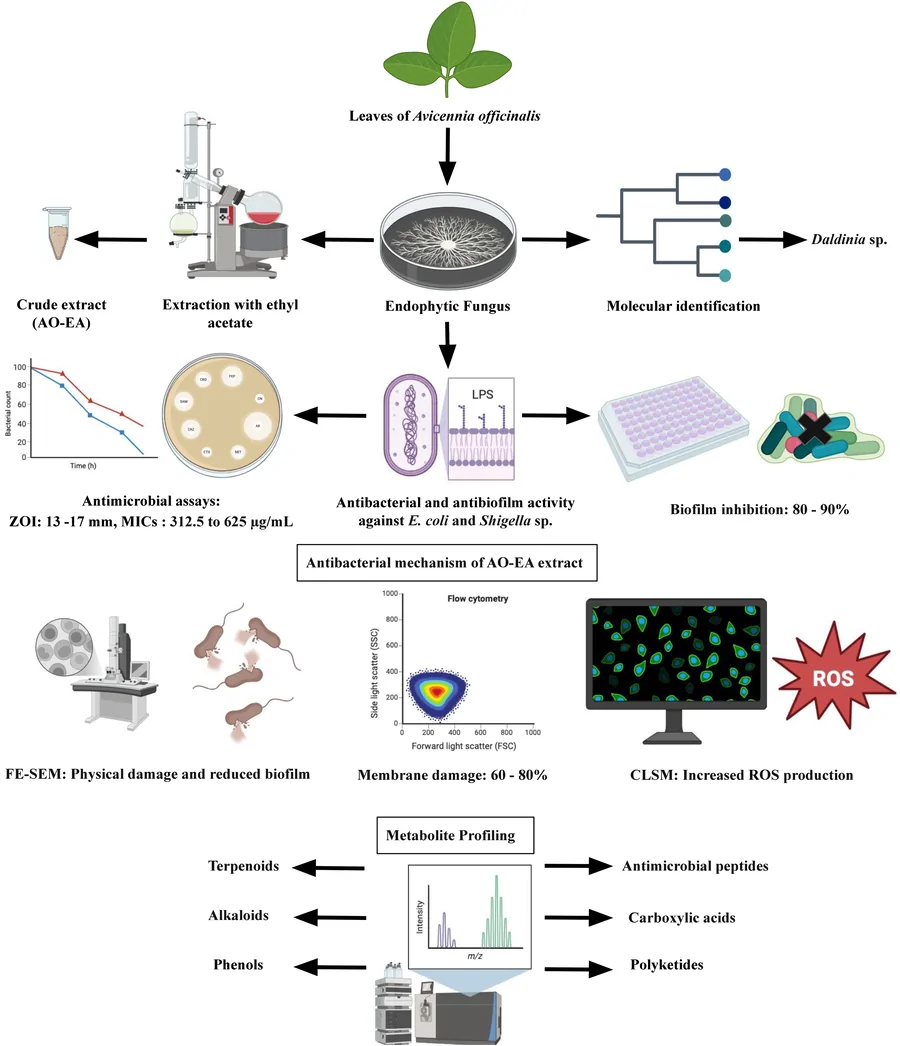

## 1 Introduction

Mangroves inhabit estuarine zones and constitute structurally and functionally complex marine habitats (Deshmukh et al. 2020). The unique position at the land‐sea interface exposes them to fluctuating salinity, tides, and nutrient regimes, creating a distinctive selective environment for both plants and associated microorganisms (Lather et al. 2026). Consequently, mangrove ecosystems have emerged as valuable reservoirs of novel genetic and metabolic diversity with important ecological, pharmaceutical, and biotechnological applications (Dahibhate et al. 2019). Mangrove plants harbor a wide variety of endophytic microorganisms, including bacteria and fungi that live within plant tissues without causing any harm (Wilson 1995). These microorganisms generally remain asymptomatic and persist within their host plants, where they can promote plant growth and improve tolerance to biotic and abiotic stresses while receiving nutrients and protection from the host (M. Jia et al. 2016). Endophytic fungi, in particular, can colonize plants for either part or the entirety of their life cycle without producing visible symptoms of disease (Kusari et al. 2012). The challenging conditions of mangrove environments, including high salinity, tidal fluctuations, and variations in oxygen availability, have contributed to the development of specialized metabolic pathways in these endophytes. As a result, they can produce a broad range of secondary metabolites with novel chemical structures and biological activities (Agrawal and Bhatt 2023), including antibacterial, anti‐inflammatory, antioxidant, and anticancer properties (Harikrishnan et al. 2021). Mangrove‐associated endophytic fungi, such as *Aspergillus*, *Penicillium*, *Colletotrichum*, and *Schizophyllum*, are taxonomically and functionally diverse group capable of producing phenolics, terpenoids, alkaloids, antimicrobial peptides, and other bioactive metabolites (Yang et al. 2024). Many of these metabolites show strong antibacterial activity against pathogenic bacteria and may therefore serve as promising candidates for antibiotic discovery (Bansal et al. 2026). Their antimicrobial effects can occur through different mechanisms, such as membrane disruption, protein denaturation, enzyme inhibition, induction of oxidative stress, modulation of efflux pumps, and quorum‐sensing suppression (Ali et al. 2024). Taken together, these findings indicate that mangrove plants harbor a diverse range of biologically active endophytic fungi that could be explored for the discovery of novel antimicrobial compounds.

Resistance to antimicrobial and chemotherapeutic drugs has become a major global health concern (Kumari et al. 2026). According to recent estimates from the World Health Organization (WHO), antimicrobial resistance (AMR) is a major contributor to mortality worldwide. In 2021, bacterial AMR was associated with approximately 1.14 million deaths (World Health Organization 2025). Moreover, between 1990 and 2021, AMR‐associated deaths among adults aged 70 years and older increased by more than 80%. Projections indicate that by 2050, AMR could be directly responsible for approximately 1.91 million deaths and be associated with a further 8.22 million deaths globally (Naghavi et al. 2024). The misuse and overuse of antibiotics have further accelerated the spread of AMR among critical pathogens (Murray et al. 2022). These trends highlight the need for more responsible antibiotic use as well as the development of alternative antimicrobial agents to address the growing threat of AMR. The problem affects both Gram‐positive and Gram‐negative bacteria, many of which are responsible for severe infections in community and healthcare settings. Gram‐negative pathogens, including *Escherichia coli*, *Acinetobacter baumannii*, *Pseudomonas aeruginosa*, and *Shigella* sp., possess a structurally dynamic cell envelope that promotes antibiotic resistance and contributes to increased mortality. Their complex cell envelopes and multiple resistance mechanisms, such as antibiotic‐inactivating enzymes, porin remodeling, efflux pumps, and altered drug targets, render many antibiotics ineffective (Gauba and Rahman 2023).

The most common and dominant form of microbial growth in nature is biofilms. Biofilms are microbial communities that produce an extracellular matrix (Flemming et al. 2016). They create diverse environmental and clinical conditions that confer substantial advantages on pathogenic bacteria by increasing their tolerance to antibiotics and host immune responses (Muhammad et al. 2020). The extracellular matrix, composed of polysaccharides, proteins, nucleic acids, and lipids (González‐Ramírez et al. 2016), supports efficient nutrient uptake, facilitates cell–cell communication, and enables secretion and retention of extracellular enzymes (Gupta et al. 2016). Many important pathogens, including *E. coli* and *Shigella* sp., form robust biofilms (Hassuna et al. 2024; Evangeline et al. 2024), which markedly enhance AMR through mechanisms, such as multidrug efflux pumps, reduced envelope permeability, restricted antibiotic penetration, type IV secretion systems, and antibiotic‐modifying enzymes (Hall and Mah 2017). Therefore, new antibacterial agents capable not only of killing planktonic cells but also of preventing biofilm formation or disrupting established biofilms are critically needed (Belay et al. 2024). These trends collectively underscore the urgent need for new, effective antimicrobials, particularly those with activity against multidrug‐resistant pathogens and biofilm‐associated infections.

Bioactive metabolites produced by mangrove‐associated endophytic fungi are increasingly being explored as potential alternatives for treating drug‐resistant infections. The extreme and continuously changing conditions of mangrove environments may encourage these fungi to produce chemically diverse and novel metabolites (Debbab et al. 2013). In addition, their close association with plant hosts in an environment where microorganisms are constantly competing with one another may contribute to the production of compounds with broad‐spectrum antibacterial and antibiofilm activities (Nazir et al. 2024). Several studies have reported the antimicrobial potential of endophytic fungi isolated from mangrove plants. For example, Buatong et al. (2011) reported diverse endophytic fungal communities associated with *Avicennia officinalis* and other mangrove plants, highlighting their ability to produce a variety of bioactive compounds. Joel and Bhimba (2013) isolated the endophytic fungus *Schizophyllum commune* from *A. officinalis* and evaluated the antibacterial activity of its ethyl acetate extract. The extract showed activity against important pathogens, including *Vibrio cholerae*, *Micrococcus luteus*, and *Staphylococcus aureus*. Similarly, fumigaclavine C, azaspirofuran B, and fraxetin were isolated from the endophytic fungus *Aspergillus fumigatus* associated with leaves of *Ceriops decandra*. These compounds exhibited strong bioactivity against *S. aureus*, *M. luteus*, *E. coli*, and *P. aeruginosa* (Zihad et al. 2022). In another study, *Aspergillus niger* was the predominant fungal endophyte associated with the mangrove species *Rhizophora mucronata*, *Bruguiera gymnorrhiza*, *Avicennia marina*, *Xylocarpus granatum*, and *Heritiera littoralis*, showing antibacterial activity against *S. aureus*, *E. coli*, and *P. aeruginosa* (Wacira et al. 2024).

Many mangrove plants have yet to be systematically investigated for their associated endophytic communities. Even when endophytes have been successfully isolated, information on their metabolic pathways and the bioactive compounds they produce is often limited. Moreover, most of the available studies have concentrated mainly on antibacterial activity against planktonic cells, while relatively few have examined antibiofilm activity, underlying mechanisms of action, and the characterization of the compounds responsible for these effects. This knowledge gap is particularly noticeable in mangrove species, such as *A. officinalis*, which remains relatively unexplored in regions, such as Goa, India, despite its ecological importance and traditional medicinal relevance. The present study, therefore, focused on fungal endophytes isolated from *A. officinalis* collected from the mangrove ecosystems of Goa, with the aim of addressing some of these gaps. A fungal endophyte was isolated and identified, and its ethyl acetate extract was examined for antibacterial and antibiofilm activity against selected pathogens. The possible mechanisms underlying these activities were also investigated, with particular focus on membrane integrity, reactive oxygen species (ROS) generation, and biofilm inhibition or disruption. In addition, high‐resolution liquid chromatography–mass spectrometry (HR‐LCMS) analysis was carried out to identify the bioactive metabolites present in the extract. To our knowledge, this study represents the first report of antimicrobial and antibiofilm activity from a fungal endophyte associated with *A. officinalis* in Goa.

## 2 Materials and Methods

### 2.1 Isolation of Mangrove Endophytes

Healthy and freshly collected leaves of *A. officinalis* were obtained from Chorao Island, Goa, India, and used for the isolation of fungal endophytes. The collected leaves were transferred into sterile plastic bags, appropriately labeled, and brought to the laboratory for further processing. The samples were first washed thoroughly under running tap water and then rinsed twice with distilled water to remove adhering debris and surface‐associated microorganisms. Surface sterilization was subsequently carried out following the standard protocol described by Airin et al. (2024), with minor modifications. Briefly, the leaves were immersed in 70% ethanol (v/v) for 60 s and then treated with 4% sodium hypochlorite (w/v) for 2 min. The treated leaves were rinsed 5–6 times with autoclaved distilled water and then dried under sterile conditions. After sterilization, the leaves were aseptically cut into small segments, and 4–5 segments were placed on potato dextrose agar (PDA; HiMedia) supplemented with 1% tartaric acid (HiMedia), streptomycin (50 μg/mL; HiMedia), and chloramphenicol (50 μg/mL; HiMedia). The antibiotics were included to suppress bacterial growth and facilitate the recovery of fungal endophytes. The plates were incubated at 28°C ± 2°C for 7–21 days and monitored periodically for fungal growth. The efficiency of the surface sterilization procedure was checked by plating the final washings. Sterilization was considered effective when no microbial colonies developed from the washings, while fungal hyphae emerged only from the cut edges of the leaf segments. The emerging fungal endophytes were periodically transferred to fresh PDA plates to obtain pure cultures of individual fungal isolates. These isolates were maintained on PDA plates at 4°C and in 50% glycerol at −80°C (Sopalun et al. 2021).

### 2.2 Morphological and Molecular Identification of Fungal Endophyte

Endophytic fungi were initially identified based on morphological characteristics. After incubation, colony features, growth patterns, and colony color were recorded. Spores and mycelia from purified colonies were mounted on glass slides, stained with lactophenol cotton blue (1% v/v; HiMedia), and examined under a light microscope. In addition, spores and hyphae were observed using a field‐emission scanning electron microscope (FE‐SEM; FEI, Quanta 250 FEG, 30 kV) (Shamsudin et al. 2025). Selected isolates producing valuable bioactive compounds were further identified using molecular methods. Isolated colonies were suspended in 50 μL of molecular biology‐grade water and mixed thoroughly. The suspension was heated at 95°C for 10 min and briefly centrifuged. The supernatant served as the polymerase chain reaction (PCR) template with universal primers ITS1 and ITS4, targeting the internal transcribed spacer (ITS) region (Martin and Rygiewicz 2005). The PCR product was then subjected to cycle sequencing using BigDye Terminator v3.1 (BDT v3.1) chemistry and subsequently sequenced on an Applied Biosystems (ABI) 3500XL Genetic Analyzer. Additional internal primers were used to obtain a near‐full‐length sequence, yielding high‐quality base reads covering the target. The .ab1 trace files obtained after sequencing were manually curated, assembled into a contiguous sequence, and exported as a FASTA file. The consensus sequence was subsequently subjected to database search against the Unite 9.0 (Abarenkov et al. 2010) using the Basic Local Alignment Search Tool (BLAST) (Altschul et al. 1990). For phylogenetic analysis, up to 20 closest‐neighbor sequences from different taxa were selected from the top 1000 BLAST hits based on similarity from the database National Center for Biotechnology Information (NCBI) and aligned with MUltiple Sequence Comparison by Log‐Expectation (MUSCLE) (Edgar 2004). The alignment was manually inspected and used to construct a consensus phylogram via maximum likelihood or neighbor‐joining with 1000 bootstrap iterations (Nguyen et al. 2014) in MEGA12.

### 2.3 Extraction of Bioactive Compounds

Bioactive compounds were extracted using the methods described by Nurunnabi et al. (2020), with key modifications to improve the results. Briefly, the isolated fungal strain was inoculated into 250 mL conical flasks containing 100 mL potato dextrose broth (PDB) and incubated at 28°C ± 2°C in the dark with constant shaking at 100 rpm for 21 days. This incubation period was necessary to facilitate the extracellular (secreted) secondary metabolites produced by the endophyte in PDB (Singh and Kumar 2023). After incubation, the culture broth was separated from the mycelium by filtration through Whatman No. 1 filter paper. The filtrate was extracted with organic solvents, such as ethyl acetate and methanol (1:1, v/v; Finar Chemicals), and the mixture was kept at room temperature for 48 h to allow complete solubilization of fungal metabolites. The mixture was then concentrated under reduced pressure at 40°C–45°C using a rotary evaporator (Heidolph) to obtain the crude extract. The crude extracts were weighed and stored at −20°C until further use. The concentrated fungal metabolites were reconstituted in dimethyl sulfoxide (DMSO; 1% v/v) at various concentrations (10 and 20 mg/mL) and filtered through 0.2 μm membrane filters (HiMedia) for antimicrobial testing. Among the two extracts, methanol or ethyl acetate, the more active extract was taken forward for further experiments.

### 2.4 Agar Well Diffusion Assay

The fungal extract was screened against two reference Gram‐negative pathogenic bacterial strains, namely, *E. coli* (MTCC 723) and *Shigella* sp. Microorganism working cultures for investigations were made fresh by adding stock cultures to 5 mL of Mueller–Hinton broth (MHB; HiMedia) and growing at 37°C for 16–18 h at 120 rpm. Fungal extracts from the isolates were subjected to an agar well diffusion assay to determine their antimicrobial activities against the above‐mentioned pathogenic bacteria, using extracts obtained with different solvents as described by Dahibhate et al. (2020). A bacterial inoculum of 1.0 × 10^8^ CFU/mL (colony‐forming units/mL) was prepared using sterile saline (0.9% w/v). The suspensions were uniformly spread on Mueller–Hinton agar (MHA) plates. Wells with a diameter of 6 mm were punched aseptically using a sterile cork borer, and 100 μL of the crude extracts at concentrations of 10 and 20 mg/mL were added. Ciprofloxacin (8 μg/mL; HiMedia) was used as the positive control and 1% v/v DMSO as the negative control. The culture plates were incubated at 37°C for 16–18 h. Antimicrobial activity was evaluated by measuring the diameter of clear zones of inhibition using a standard zone scale (HiMedia). All assays were performed in triplicate.

### 2.5 Minimum Inhibitory Concentration (MIC) Assay and Minimum Bactericidal Concentration (MBC) Assay

MIC is the lowest concentration of an antimicrobial agent that prevents visible growth of a microorganism in vitro. MIC of fungal extracts against the tested bacterial pathogens was determined by broth microdilution, following Clinical and Laboratory Standards Institute (CLSI 2015b) guidelines. Fungal extracts were serially diluted twofold in MHB in sterile 96‐well plates (Tarsons) to obtain final concentrations of 10, 5, 2.5, 1.25, 0.625, 0.3125, and 0.156 mg/mL. A bacterial suspension (1.0 × 10^6^ CFU/mL) was added to each well, and the plates were incubated at 37°C for 16 h. Ciprofloxacin (8 μg/mL) was used as the positive control, 1% (v/v) DMSO with bacterial culture as the negative control, and MHB alone as the medium control. Following incubation, the optical density (OD) of each well was measured at 600 nm using a ultraviolet–visible (UV/Vis) multiplate spectrophotometer (ThermoScientific). To determine the MBC, 10 μL of culture from each well showing no visible growth was streaked onto MHA plates. The MBC was defined as the lowest extract concentration that failed to show visible growth on agar plates after incubation at 37°C for 16 h. All assays were conducted in triplicate.

### 2.6 Time‐Kill Kinetics Assay

A time‐kill kinetics assay is an in vitro method used to assess the rate at which an antimicrobial agent reduces a microorganism's viability over time. Time‐kill kinetics were analyzed using MIC values, following the method chosen by Israyilova et al. (2022) with some modifications. In all, 1% v/v of a 24‐h bacterial culture was transferred to 5 mL of freshly prepared sterile MHB and incubated for 2–3 h at 37°C. The culture was then diluted with sterile MHB to obtain a starting inoculum of approximately 1.0 × 10^5^–1.0 × 10^6^ CFU/mL. The cells were exposed to fungal extract at MIC. Samples were collected at 0, 6, 12, 18, and 24 h. From each tube, a 100‐μL aliquot was transferred to a tube containing 900 μL of sterile MHB, and the mixture was then serially diluted in MHB. Then, 100 μL of each dilution was spread‐plated onto sterile MHA plates. The plates were incubated at 37°C for 24 h. An inoculum without fungal extract served as the growth control, while MHB was included as a quality control check. The results were expressed as mean log_10_ CFU/mL, and time‐kill curves were generated by plotting log_10_ CFU/mL against time. Additionally, 100 μL of culture was used to measure turbidity using a 96‐well UV–Vis microplate spectrophotometer. The OD_600_ of the bacterial suspensions was measured at various time intervals (0, 4, 8, 16, and 24 h), and OD was plotted against time. All assays were performed three times.

### 2.7 Field‐Emission Scanning Electron Microscopy

FE‐SEM was employed to assess the impact of the crude bioactive secondary metabolite on bacterial cells. For this procedure, we employed the methodologies outlined by Lin et al. (2023), incorporating minor modifications. Briefly, 1% (v/v) of a 24‐h bacterial culture was added to 5 mL of freshly prepared sterile MHB and incubated for 2–3 h at 37°C. Cells were treated with fungal extract at concentrations matching their MICs and incubated for 3 h at 37°C. An inoculum without fungal extract served as the growth control. After incubation, both treated and untreated bacterial cells were collected by centrifugation (Eppendorf) at 3000 rpm for 10 min at 4°C. Cell pellets were washed twice with sterile 1x PBS (Phosphate Buffered Saline) and resuspended in 1 mL of 1x PBS. A 20‐μL sample of bacterial suspension was smeared onto a 12‐mm coverslip and air‐dried. Samples were fixed with 2.5% (v/v) glutaraldehyde (HiMedia) in 0.1 M sodium phosphate buffer (pH 7.2) overnight at 4°C, then washed twice with 1x PBS. Bacterial cells were dehydrated with graded ethanol (30%–100%) for 5 min at each concentration. The final concentrations were used for complete drying. The samples were mounted on aluminum stubs, sputter‐coated with gold, and examined with an FE‐SEM at various magnifications. The process was repeated three times.

### 2.8 Flow Cytometry Analysis

Flow cytometry with PI (Sigma‐Aldrich) staining was utilized to assess cell membrane integrity. The methodology described by Pandy et al. (2023) was used, with slight modifications. A 1% (v/v) portion of a 24‐h bacterial culture was inoculated into 5 mL of freshly prepared sterile MHB and incubated for 2–3 h at 37°C. The cells were then exposed to fungal extract at 2x MIC and incubated for an additional 3 h at 37°C. An inoculum without fungal extract served as the growth control, and another tube containing cells treated with 70% ethanol served as the positive control. After incubation, cells were centrifuged, resuspended in 1 mL of 1x PBS, and 10 μL of PI (Stock: 1 mg/mL) was added, followed by incubation at 37°C in the dark for 15 min. After this, the cells were centrifuged again, suspended in 1 mL of 1x PBS, and transferred to flow cytometry tubes for analysis using a FACScan flow cytometer (BD Biosciences, FACS Melody). The procedure was repeated in three independent runs.

### 2.9 Confocal Scanning Laser Microscopy (CSLM)

CSLM is a high‐resolution, three‐dimensional imaging technique used to evaluate the antimicrobial effects of extracts on bacterial cells by assessing membrane integrity and viability (Shahina et al. 2022). For sample preparation, we employed the methodology outlined by Gu et al. (2023) with some modifications. In this experiment, 1% (v/v) of a 24‐h bacterial culture was inoculated into 5 mL of freshly prepared sterile MHB and incubated for 2–3 h at 37°C. The culture was then treated with fungal extract at 2x MIC and incubated for an additional 3 h at 37°C. A culture without fungal extract served as the growth control, while cells treated with H_2_O_2_ and 70% ethanol served as the positive control. Cells were harvested by centrifugation at 3000 rpm for 10 min at 4°C, and the pellets were washed twice with 1x PBS or sterile saline (0.9% NaCl) to remove remaining nutrients and unbound extract. The pellets were then resuspended and incubated with fluorescent dyes [PI and dichlorodihydrofluorescein diacetate (DCFDA)] in the dark for 15–30 min at room temperature or 37°C. Following staining, the cells were centrifuged again and resuspended in 1 mL of 1x PBS to remove excess dye. A 20 μL aliquot of the stained cell suspension was placed on a clean confocal disk. Images were acquired three times using an LSM710 inverted confocal laser‐scanning microscope (Olympus FV3000).

### 2.10 Antibiofilm Assay

Biofilm formation assays were performed in sterile 96‐well plates to quantify reductions in biomass and metabolic activity. For biofilm assessment, 5 mL of MHB was inoculated with a freshly grown 24‐h culture and incubated at 37°C with shaking at 120 rpm for 18–20 h. Cells were centrifuged, washed twice with sterile 1x PBS, and resuspended in MHB. Two biofilm stages were evaluated: preventing initial attachment and disrupting a preformed biofilm after 24 h. Biofilms were allowed to develop for 0 h (T0) or 24 h (T24) before extracts were added at the MIC. For T0, 20 μL of standardized culture (1.0 × 10^6^ CFU/mL in MHB) was added to sterile 96‐well plates, followed by 90 μL of extract and 90 μL of MHB, and incubated for 24 h at 37°C without shaking. For T24, 200 μL of standardized culture was incubated for 24 h to allow biofilm formation before the addition of extract. For both T0 and T24, the following controls were included: growth control (culture + MHB), positive control (culture + MHB + antibiotic [ciprofloxacin]), treated (extract + MHB), negative control (culture + 1% DMSO + MHB), and medium control (MHB only). After 24 h, a modified crystal violet staining (CVS) assay and a 3‐(4,5‐dimethylthiazol‐2‐yl)‐2,5‐diphenyltetrazolium bromide (MTT) assay were performed to quantify reductions in biofilm biomass and metabolic activity (Olawuwo et al. 2022).

### 2.11 Crystal Violet Staining

Both live and dead cells are stained with crystal violet. The total biofilm biomass was evaluated using CVS. The biofilm inhibition assay was carried out as described by Lacotte et al. (2022), with some modifications. Following incubation as described above, the wells were carefully emptied and washed at least three times with sterile 1x PBS to remove unattached or loosely attached cells, and then dried for 30 min. Subsequently, biofilms were stained with 200 μL of 0.1% (w/v) aqueous crystal violet (Sigma‐Aldrich) for 20 min at room temperature. Excess stain was removed by washing the wells at least three times with 1x PBS, and 200 μL of 96% ethanol was added to each well to resolubilize the crystal violet bound to adherent cells. After 10 min, absorbance was recorded at 570 nm (OD_570_) using a microplate reader, and results were expressed as percentage inhibition using the equation below. Three independent replicates were conducted.

$$\text{Percentage}\left(\%\right)\;\text{inhibition}=\frac{{(\text{OD}}_{\text{growth}\;\text{control}}-{\text{OD}}_{\text{Sample}})\times 100}{{\text{OD}}_{\text{growth}\;\text{control}}}.$$

### 2.12 MTT Assay

MTT (HiMedia) is a yellow compound that is reduced by dehydrogenases in viable cells to form the purple formazan. The intensity of this purple color, which reflects the number of viable cells, was measured spectrophotometrically. MTT assay was performed with some modifications (Kulišová et al. 2023). Briefly, after removing the cell suspension from the microtiter plate wells, the biofilm‐containing wells were washed twice with 1x PBS. To assess metabolically active cells within the biofilm, MTT solution (0.5 mg/mL) was added to each well. The plate was incubated on a shaker in the dark for 3 h (37°C, 120 rpm). Then, 200 μL of DMSO was added to dissolve the purple formazan crystals generated by metabolic activity. The plate was incubated again on a shaker in the dark for 30 min (37°C and 120 rpm). Absorbance was recorded at 570 nm to monitor the colorimetric change. Three independent replicates were conducted.

The biofilm‐inhibitory effect was calculated as the percentage of biofilm viability relative to untreated controls using the following equation:

$$\text{Percentage }(\%)\;\text{Viability}=\frac{{\text{OD}}_{570\;\text{treated}}}{{\text{OD}}_{570\;\text{growth}\;\text{control}}}\times 100.$$

### 2.13 Field‐Emission Scanning Electron Microscopy of Biofilm Formation

Bacterial biofilm formation and morphological changes in mature biofilms treated with fungal extracts were analyzed using FE‐SEM. For the assay, 2 mL of MHB broth was added to culture tubes containing sterilized coverslips (12 mm diameter), followed by 20 μL of an overnight bacterial culture. The tubes were incubated for 48 h at 37°C. An inoculum without fungal extract served as the growth control. After biofilm formation, fungal extracts at their respective concentrations were added, and the cultures were incubated without agitation at 37°C for 6 h. Following treatment, the biofilms were rinsed twice with sterile 1x PBS and air‐dried. The samples were then fixed with 2.5% glutaraldehyde overnight at 4°C, followed by two washes with 1x PBS. The attached cells were dehydrated in a graded ethanol series (30%–100%) for 5 min at each step. Finally, the samples were mounted on aluminum stubs, sputter‐coated with gold, and examined under a scanning electron microscope (SEM) at various magnifications in triplicate (Dahibhate et al. 2022).

### 2.14 Metabolite Screening by Liquid Chromatography With High‐Resolution Orbitrap Mass Spectrometry (LC‐HRMS) Orbitrap Analysis

The secondary metabolites of the active extract were analyzed qualitatively using an HR‐LCMS (LC‐HRMS) Orbitrap. The specific instrument employed was the Q‐Exactive Plus Biopharma, paired with a Hypersil Gold column (150 × 2.1 mm, 1.9 μm) from ThermoScientific. For the analysis, two mobile phases were utilized: Solvent A, which contained 0.1% formic acid in Milli‐Q water, and Solvent B, acetonitrile. The analysis was conducted under gradient conditions at a flow rate of 300 μL/min, with linear gradient elution as follows: 5% B from 0 to 2 min, then 95% B from 2 to 20 min, and finally returning to 5% B from 21 to 30 min. The entire run lasted 30 min. For preparation, the sample was dissolved in 1 mL of Milli‐Q water to obtain a concentration of 1 mg/mL, and the mixture was mixed thoroughly to ensure complete dissolution. The solution was then filtered through a 0.22‐μm nylon membrane filter (HiMedia) before being injected. An injection volume of 5 μL was used for the analysis. Scanning data were collected in both positive and negative modes at a resolution of 70,000, covering a mass‐to‐charge ratio (*m*/*z*) range from 85 to 1100. Data acquisition was performed using Thermo Scientific Xcalibur (Version 4.2.28.14). In contrast, data processing was performed with Compound Discoverer 2.1 SP1 (Thermo Fisher Scientific), which is designed for qualitative metabolites analysis and uses accurate mass and mass spectral library searches to identify small molecules. The mass tolerance was set to 5 ppm. To identify metabolites, data were processed using compound databases such as mzCloud to confirm MS/MS fragmentation patterns and ChemSpider to validate the identified chemical structures.

### 2.15 Statistical Analysis

All experiments were performed in triplicate, and the findings were shown as mean ± standard deviation (*n* = 3). Data were analyzed using GraphPad Prism software (Version 11.0.2). All experiments were statistically analyzed using either a one‐way or a two‐way ANOVA test, as the data were normally distributed. A value of *p* < 0.05 was considered to indicate a significant difference between groups.

## 3 Results

### 3.1 *Daldinia* sp. Was Isolated From the *A. officinalis* Leaf

The morphological features of the isolated endophytic fungus are illustrated in Figure 1A–C. The colony exhibits a gray to greyish‐green color. Its surface texture varies from velvety to powdery, and certain areas appear slightly white and cottony. Overall, the colony demonstrates dense, spreading growth that covers nearly the entire Petri plate. The surface is uneven, folded, and rugose (wrinkled), with slight ridges (Figure 1A). SEM analysis reveals a dense mycelial network, with interwoven septate and branched hyphal structures. However, distinct reproductive structures, such as conidia or conidiophores, were not visible (Figure 1B). A compound micrograph of the isolated fungus revealed septate, branched hyphae, indicative of a typical fungal structure. A conidium was observed as a small, spherical to oval structure that arose from a specialized conidiophore (Figure 1C). The endophytic fungal strain was identified at the genus level by sequencing the ITS region. The ITS region sequence analysis demonstrated a 100% match with *Daldinia* sp., as documented in NCBI. Additionally, a maximum‐likelihood phylogenetic analysis was constructed, utilizing 20 coding nucleotide sequences (Figure 1D).

**Figure 1:**
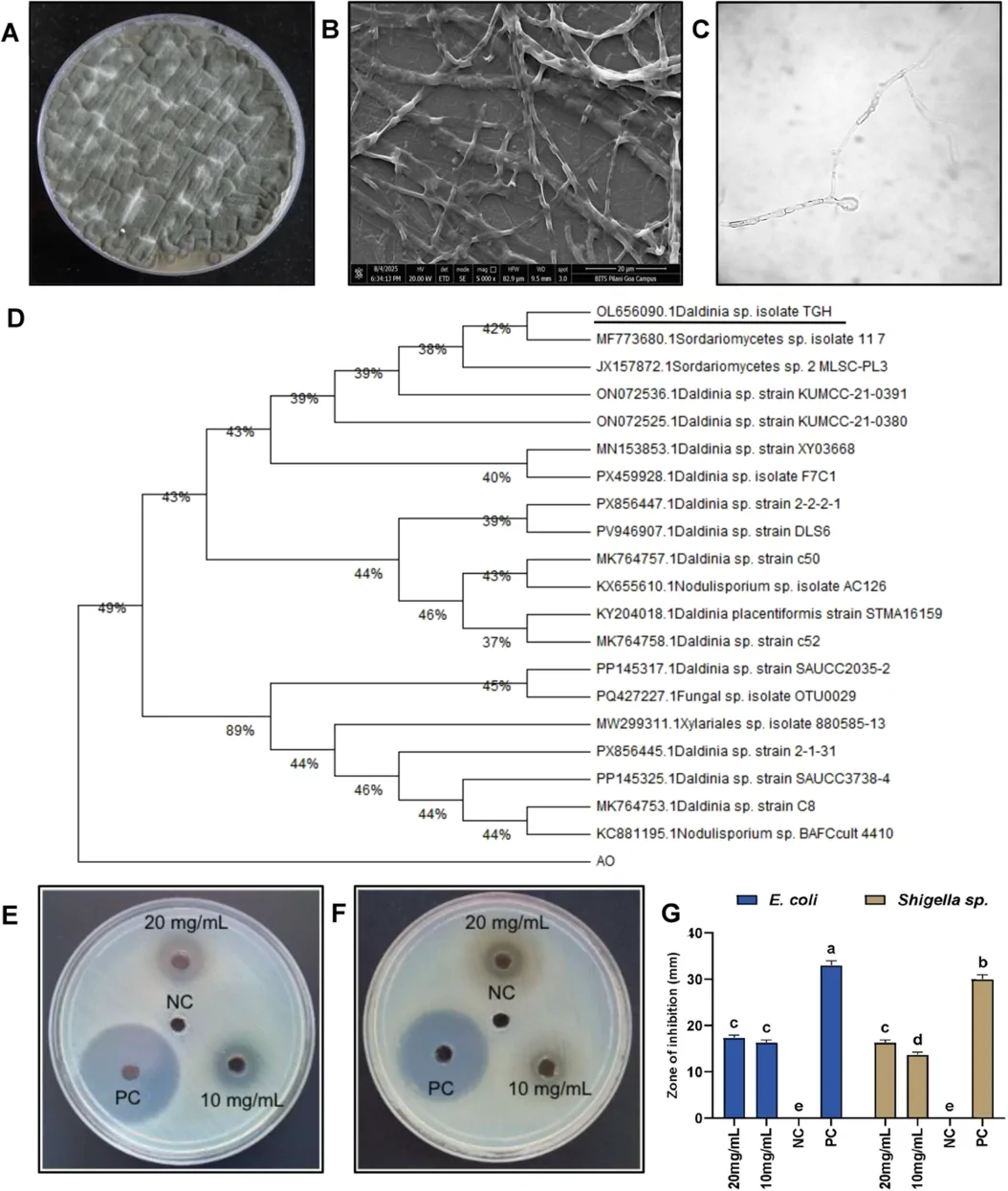
External morphology of the isolated fungal endophyte: (A) macroscopic culture on a PDA plate, (B) scanning electron micrograph, and (C) compound microscopic image. (D) Molecular identification of the isolated fungus using the universal primers ITS1 and ITS4 targeting the internal transcribed spacer (ITS) region, and phylogenetic analysis was constructed by the maximum‐likelihood method in MEGA12. AO represents the fungal endophyte isolated from *Avicennia officinalis*. Antibacterial activity of the extract AO‐EA by agar well diffusion method against (E) *Escherichia coli* and (F) *Shigella* sp. (G) Bar graph representing the zone of inhibition (mm) of AO‐EA extract at different concentrations, against *E. coli* and *Shigella* sp. DMSO, dimethyl sulfoxide; NC, negative control (1% DMSO); PC, positive control (ciprofloxacin—8 µg/mL); PDA, potato dextrose agar.

### 3.2 The Ethyl Acetate Extract Showed Potential Antimicrobial Activity Against Gram‐Negative Bacterial Pathogens

The antimicrobial assay of the extract was evaluated using an agar well diffusion assay. Among the selected solvents, ethyl acetate was found to be suitable for metabolite extraction (Figure S1 and Table S1). The ethyl acetate extract derived from the endophyte of *A. officinalis* (designated AO‐EA) demonstrated the most significant antibacterial properties against Gram‐negative bacteria, including *E. coli*, and *Shigella* sp. (Table 1). The measured inhibition zones ranged from 13.67 ± 0.57 to 17.33 ± 0.57 mm. In comparison, the inhibition zones associated with ciprofloxacin ranged from 30 ± 1 to 33 ± 1 mm. Wells incorporating 1% (v/v) DMSO exhibited no inhibition zone (Figure 1E–G).

**Table 1:** Antimicrobial activity (agar well diffusion) of the extract AO‐EA against the pathogens.

Pathogens	Zone of inhibition (mm) concentration (mg/mL)			
	20	10	PC	NC
*Escherichia coli*	17.33 ± 0.57	16.33 ± 0.57	33 ± 1	0
*Shigella* sp.	16.33 ± 0.57	13.67 ± 0.57	30 ± 1	0

Abbreviations: AO‐EA, ethyl acetate extract of *Daldinia* sp. from *Avicennia officinalis*; DMSO, dimethyl sulfoxide; NC, negative control (1% DMSO); PC, positive control (ciprofloxacin—8 μg/mL).

The AO‐EA extract exhibited an inhibitory effect, as evidenced by its MIC and MBC values presented in Table 2. The determined MIC and MBC values for the bacterial strains *E. coli* and *Shigella* sp. ranged from 312.5 to 625 μg/mL, highlighting the extract's effectiveness against these pathogenic bacteria.

**Table 2:** MIC (µg/mL) and MBC (µg/mL) of extract AO‐EA and positive control ciprofloxacin against *Escherichia coli* and *Shigella* sp.

Pathogens	AO‐EA		Ciprofloxacin	
	MIC (µg/mL)	MBC (µg/mL)	MIC (µg/mL)	MBC (µg/mL)
*E. coli*	625	625	8	8
*Shigella* sp.	312.5	625	8	8

Abbreviations: AO‐EA, ethyl acetate extract of *Daldinia* sp. from *Avicennia officinalis*; MBC, minimum bactericidal concentration; MIC, minimum inhibitory concentration.

To explore how the AO‐EA extract kills bacteria, time‐kill assays were performed over 24 h with both bacteria exposed to the crude AO‐EA extract at its MIC. Growth curves were generated by measuring absorbance at 600 nm (OD_600_) at specific time points, allowing comparison of growth patterns between cultures treated with the extract and untreated controls for both *E. coli* (Figure 2A) and *Shigella* sp. (Figure 2B). The extract suppressed bacterial growth at the MIC, demonstrating strong antibacterial activity, whereas the controls showed typical growth without inhibition at any point. In addition, time‐kill curves plotting log_10_ CFU/mL versus time showed that, at the MIC, the extract significantly reduced viable bacterial counts of *E. coli* (Figure 2C) and *Shigella* sp. (Figure 2D) over 0–24 h. These results suggest that fungal secondary metabolites produced in response to microbial infection have antibacterial effects.

**Figure 2:**
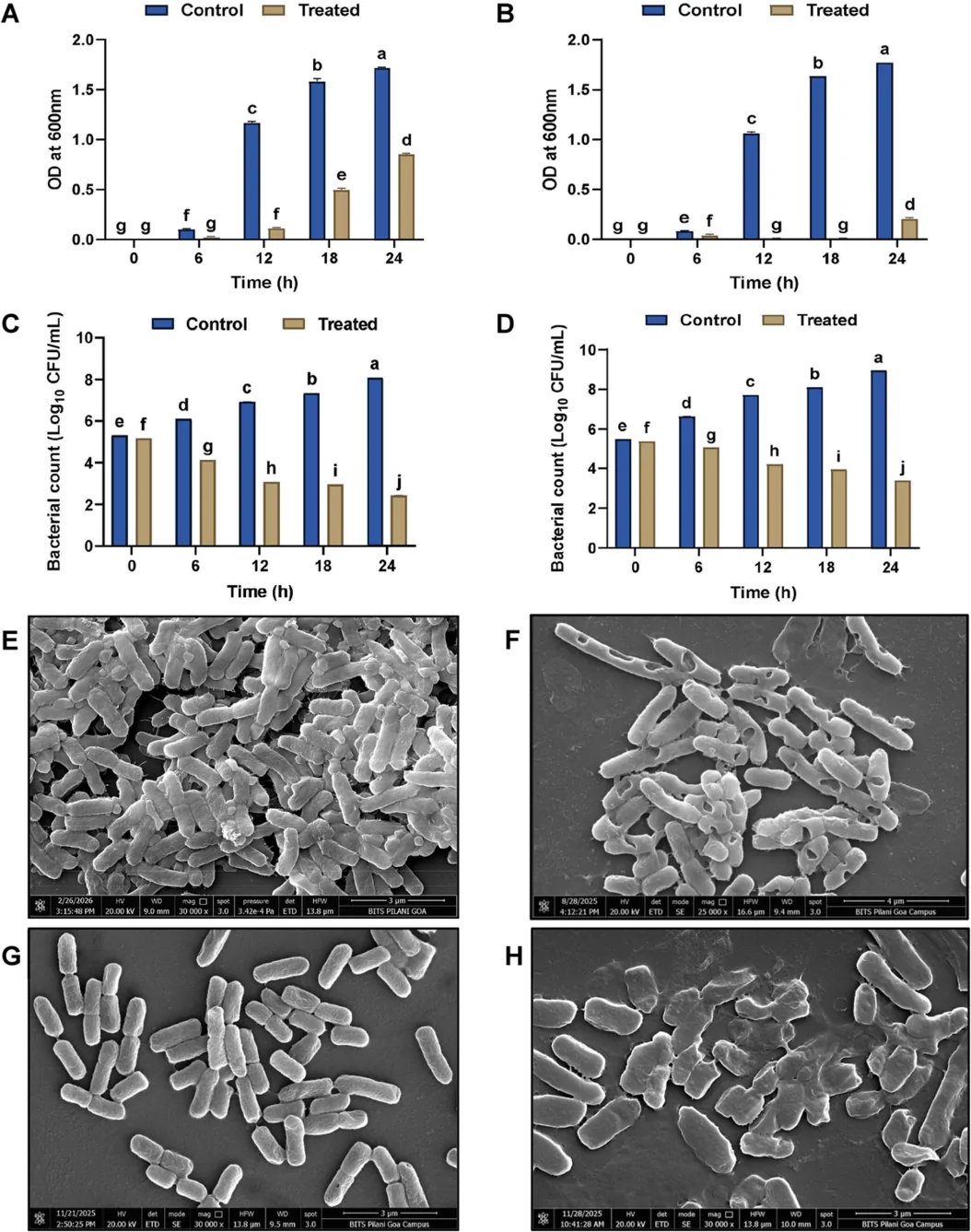
Effect of AO‐EA extract on the bacterial cell growth monitoring at the optical density (OD) at 600 nm of (A) *Escherichia coli* and (B) *Shigella* sp. with respect to time, and the viability of the (C) *E. coli* and (D) *Shigella* sp. cells with respect to time. Scanning electron micrograph of (E) untreated control of *E. coli* cells, (F) *E. coli* cells treated with AO‐EA, (G) untreated control of *Shigella* sp., and (H) *Shigella* sp. cells treated with AO‐EA at ×30,000 magnification. AO‐EA, ethyl acetate extract of *Daldinia* sp. from *Avicennia officinalis*; CFU, colony‐forming units.

### 3.3 Extract‐Induced Morphological Damage and Membrane Disruption in *E. coli* and *Shigella* sp.

The FE‐SEM analysis, over 3 h, examined morphological and physical changes in *E. coli* and *Shigella* sp., both with and without exposure to the AO‐EA extract at the MIC. As shown in Figure 2F–H, *E. coli* and *Shigella* sp. treated cells exhibit distinct structural damage induced by the extract, respectively. Compared with controls, they appear deformed and wrinkled, with some cells showing disrupted or broken parts and significantly altered shape and size. Conversely, untreated *E. coli* (Figure 2E) and *Shigella* sp. (Figure 2G) are typically rod‐shaped, with smooth, intact surfaces and continuous cell walls. Overall, the AO‐EA extract alters bacterial morphology and may inhibit growth and cause bacterial death.

We further assessed how the AO‐EA extract affects membrane permeability in *E. coli* and *Shigella* sp. using flow cytometry with PI, a red‐fluorescent dye that only enters damaged or compromised membranes. The analysis involved forward‐scatter and PI‐fluorescence signals. Cells with high PI fluorescence are classified as membrane‐damaged, while PI‐negative cells are considered viable. Fluorescence histograms of *E. coli* and *Shigella* sp. are presented in Figure 3A,C. Treated cells are found in the PI‐positive region, indicating compromised membrane integrity, while untreated cells are in the PI‐negative region, indicating intact membranes. The cell populations are displayed as dot plots of PI fluorescence versus forward scatter, with cells divided into two distinct regions. The side scatter versus PI plot demonstrates a rightward shift of the PI‐positive cells along the *x*‐axis, signifying a loss of membrane integrity due to the extract AO‐EA (Figure 3B,D). Conversely, ethanol‐treated cells have the highest number of PI‐positive events, suggesting membrane damage or cell death. After 3 h of exposure to the 2x MIC, both *E. coli* and *Shigella* sp. demonstrated a significant increase in the percentage of cells with compromised membranes, with 84.04% ± 5.89% (Figure 3A) and 62.15% ± 1.77% (Figure 3C), respectively. The percentage of PI uptake in *E. coli* and *Shigella* cells is presented in Table S2. The observed PI uptake indicates a loss of bacterial membrane integrity following treatment, suggesting that the endophytic fungal extract exerts a strong antimicrobial effect.

**Figure 3:**
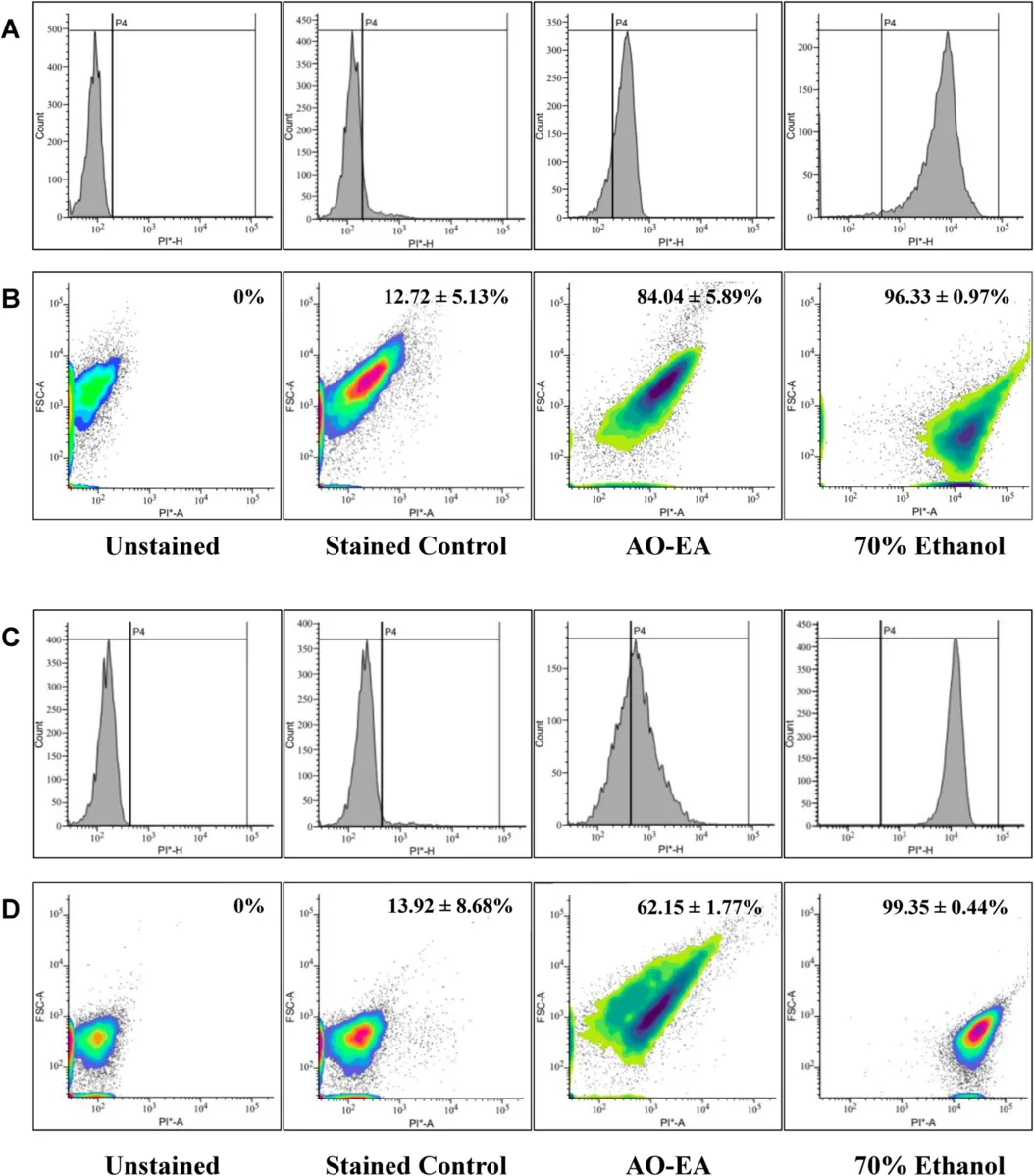
The effect of the extract AO‐EA on the cell membrane permeability of *Escherichia coli* and *Shigella* sp., determined by PI uptake. (A) Fluorescence histograms from flow cytometry analysis show a shift in the population of PI‐positive cells along the *x*‐axis for *E. coli*. (B) The side‐scatter versus PI plot shows the shift in the population of PI‐positive cells along the *x*‐axis for *E. coli*. (C) Fluorescence histograms from flow cytometry analysis show a shift in the population of PI‐positive cells along the *x*‐axis for *Shigella* sp. (D). The side‐scatter versus PI plot shows the shift in the population of PI‐positive cells along the *x*‐axis for *Shigella* sp. AO‐EA, ethyl acetate extract of *Daldinia* sp. from *Avicennia officinalis*; PI, propidium iodide.

### 3.4 AO‐EA Extract Induces ROS Production and Membrane Disruption

Excessive accumulation of intracellular ROS can cause oxidative damage to cellular biomolecules and affect membrane integrity by increasing membrane fluidity and permeability. The bacterial cells were examined using confocal laser scanning microscopy (CLSM), and fluorescence from the cell suspensions was recorded at an excitation/emission wavelength of 535/617 nm for PI. Viable bacterial cells were observed under bright‐field microscopy, while cells with damaged or compromised membranes showed red fluorescence after PI staining. Accordingly, *E. coli* (Figure 4A–C) and *Shigella* (Figure 4D–F) cells exposed to AO‐EA at the 2x MIC showed red fluorescence in the PI channel. Cells treated with 70% ethanol showed a strong fluorescent signal, indicating compromised membrane integrity in nearly all cells. Overall, these results indicate that AO‐EA disrupted the cell membrane structure, thereby increasing membrane permeability.

**Figure 4:**
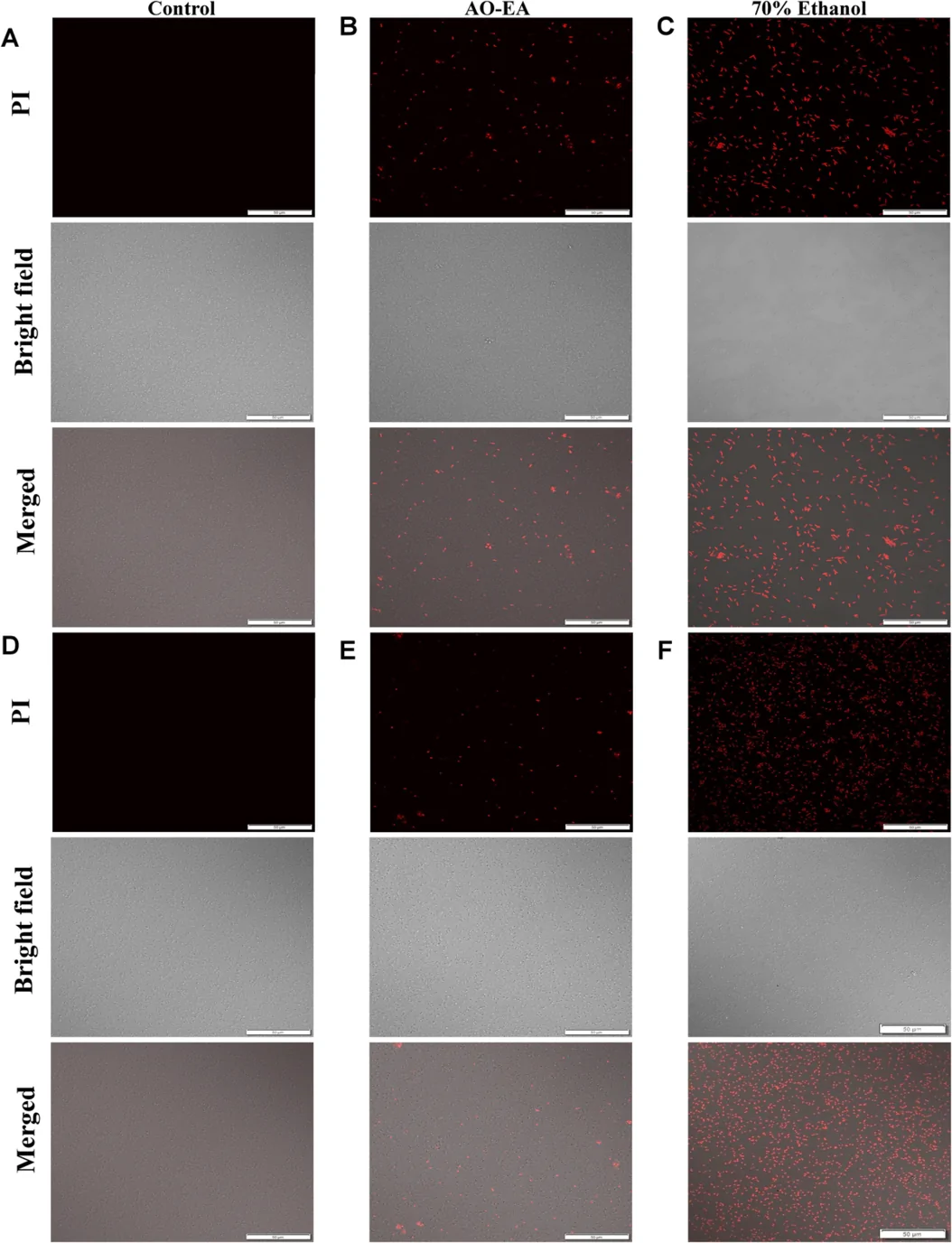
Confocal images showing cell membrane integrity of *Escherichia coli*. (A) Untreated control, (B) treated with the AO‐EA extract, and (C) treated with 70% ethanol. Confocal images showing cell membrane integrity of *Shigella* sp. (D) Untreated control, (E) treated with the AO‐EA extract, and (F) treated with 70% ethanol, monitored by PI uptake at ×60 magnification. Scale bar: 50 μm. AO‐EA, ethyl acetate extract of *Daldinia* sp. from *Avicennia officinalis*; PI, propidium iodide.

The fluorescence intensity of DCF, which is produced as a result of ROS generated by the application of AO‐EA in *E. coli* (Figure 5A–C) and *Shigella* sp. (Figure 5D–F), was visualized using CLSM. After treatment with AO‐EA at its 2x MIC, a significant increase in DCF fluorescence intensity was observed. This increase was comparable to the fluorescence levels seen with H_2_O_2_ treatment, indicating that the AO‐EA extract effectively induces ROS production in these pathogens. This suggests a potential mechanism of action for the antimicrobial effects induced by the treatment.

**Figure 5:**
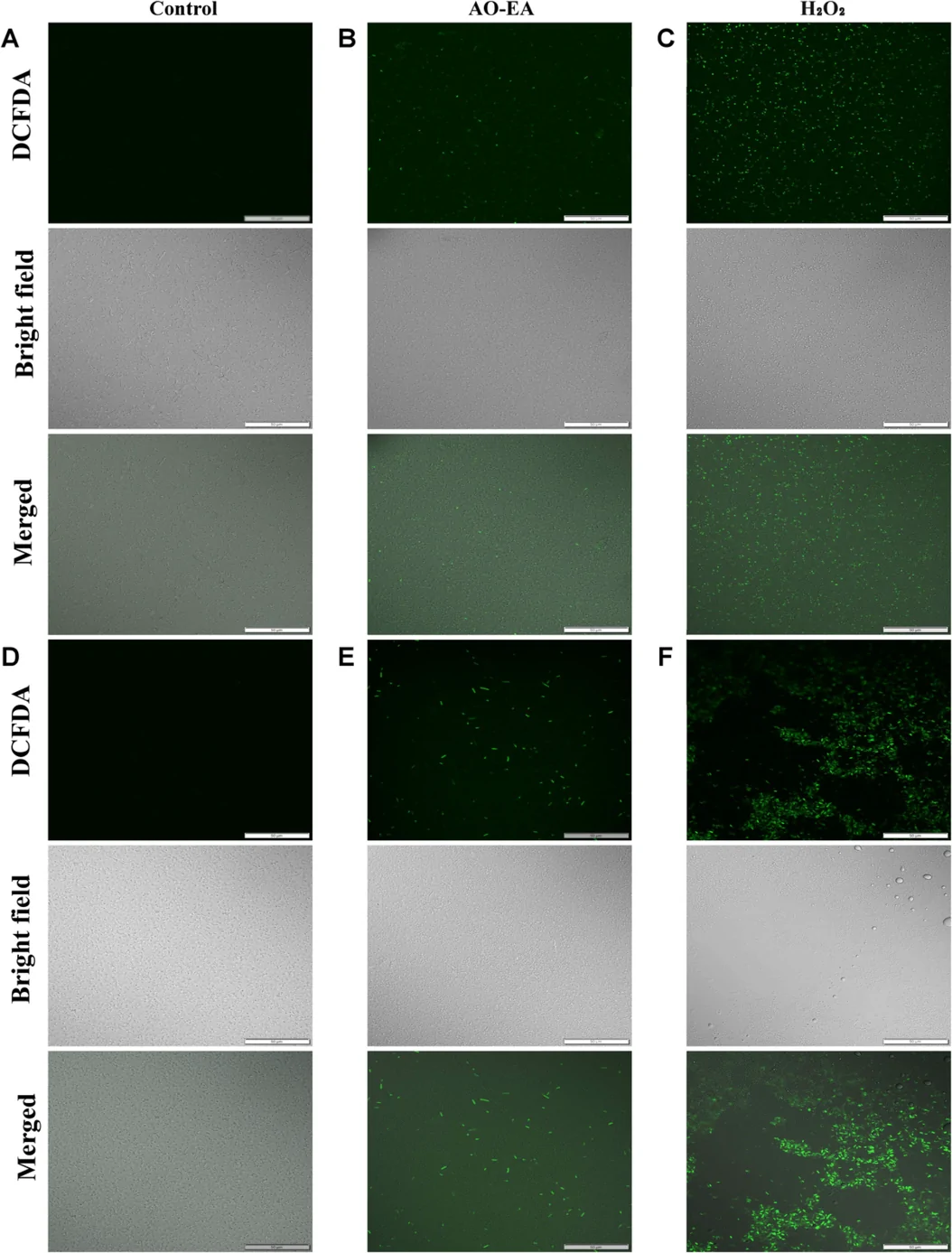
Confocal images showing ROS estimation of *Escherichia coli*. (A) Untreated control, (B) treated with the AO‐EA extract, and (C) treated with H_2_O_2_. Confocal images showing ROS estimation of *Shigella* sp. (D) Untreated control, (E) treated with the AO‐EA extract, and (F) treated with H_2_O_2_, monitored by DCFDA dye uptake at ×60 magnification. Scale bar: 50 μm. AO‐EA, ethyl acetate extract of *Daldinia* sp. from *Avicennia officinalis*; DCFDA, dichlorodihydrofluorescein diacetate; ROS, reactive oxygen species.

### 3.5 AO‐EA Extract Exhibits Antibiofilm Activity

The suppression of biofilm development at early stages is a crucial determinant of the test compound's antibiofilm efficacy. The crystal violet quantification assay evaluated the extract's efficacy in inhibiting initial biofilm formation by *E. coli* and *Shigella* sp. The results indicated that the AO‐EA extract at its MIC significantly inhibited *E. coli* biofilm formation by 93.03% ± 1.65% (Figure 6A) and *Shigella* biofilm formation by 89.38% ± 0.91% (Figure 6B). Eradication of preformed biofilms is more challenging and demands the development of highly effective alternative treatments. We therefore extended our study to evaluate the extract's ability to eradicate preformed biofilms of both *E. coli* and *Shigella* sp. strains. Before treatment, *E. coli* and *Shigella* sp. were allowed to form a robust biofilm network for 24 h. At the MIC, *E. coli* biofilm mass was reduced by up to 86.20% ± 3.35% (Figure 6C), while 83.02% ± 1.61% of *Shigella* biofilm was significantly eradicated (Figure 6D).

**Figure 6:**
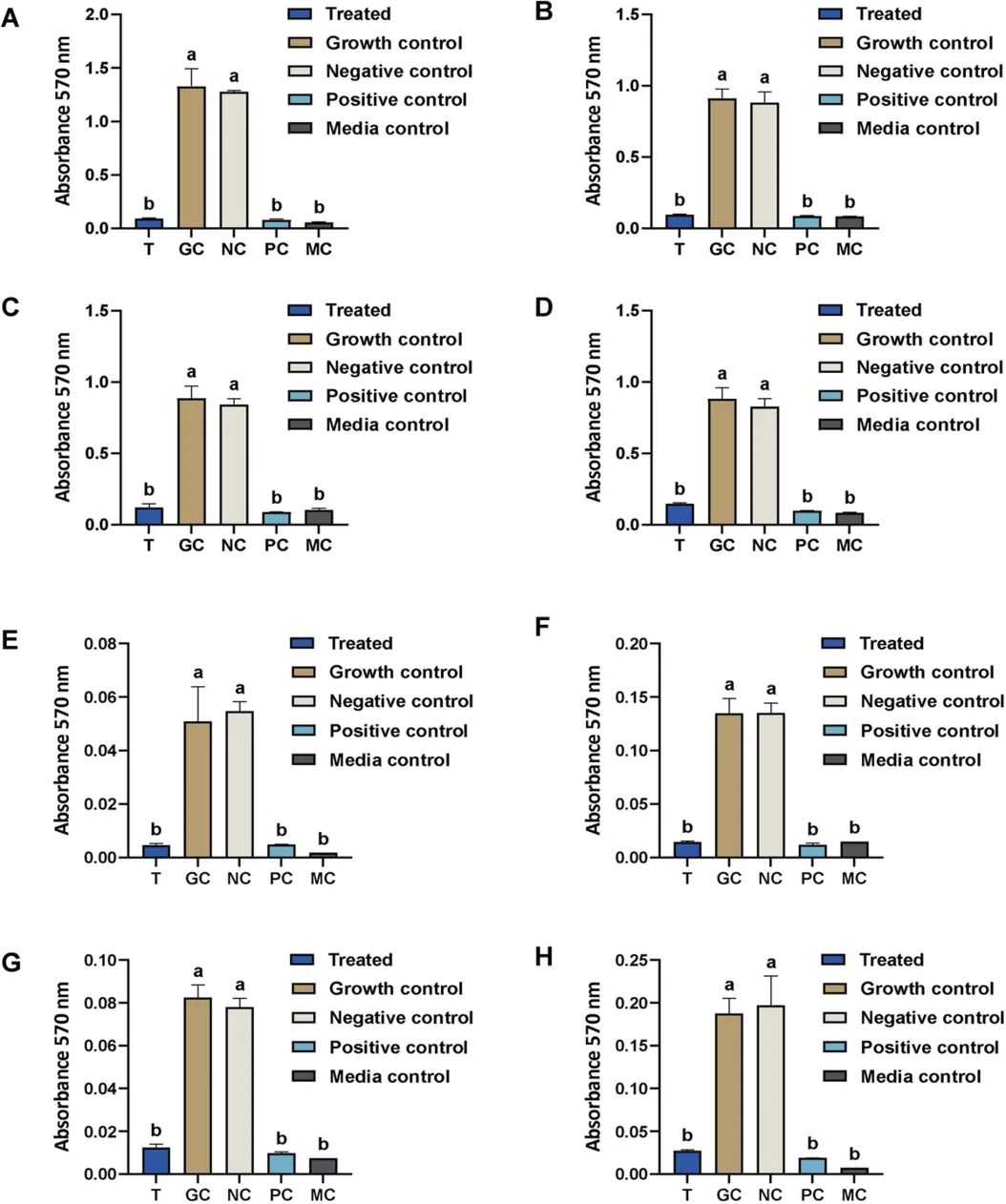
Crystal violet staining assay. Inhibition of new biofilm formation using extract AO‐EA at its MIC concentration against (A) *Escherichia coli* and (B) *Shigella* sp. Disruption of preformed biofilm using extract AO‐EA at its MIC concentration against (C) *E. coli* and (D) *Shigella* sp. MTT assay method for quantifying biofilm cell metabolic activity. Effects of AO‐EA extract on (E) *E. coli* and (F) *Shigella* sp. for the inhibition of new biofilm formation. Effects of AO‐EA extract on (G) *E. coli* and (H) *Shigella* sp. on the disruption of preformed biofilm. Positive control, ciprofloxacin:10 μg/mL; negative control: 1% DMSO. AO‐EA, ethyl acetate extract of *Daldinia* sp. from *Avicennia officinalis*; DMSO, dimethyl sulfoxide; MIC, minimum inhibitory concentration; MTT, 3‐(4,5‐dimethylthiazol‐2‐yl)‐2,5‐diphenyltetrazolium bromide.

Redox dye‐based methods, such as the MTT assay, are commonly used to measure cell viability and metabolic activity. Here, the MTT assay was used to assess the viability and metabolic activity of bacterial cells in biofilms. Results revealed a significant decline in both metabolic activity and viability of *E. coli* and *Shigella* sp. at the MIC of the AO‐EA extract. Specifically, only 10.78% ± 5.64% of *E. coli* (Figure 6E) and 11.17% ± 2.89% of *Shigella* (Figure 6F) biofilm cells were viable and metabolically active at MIC compared with the control in the early stages of biofilm formation. However, the metabolic activity and viability of *E. coli* and *Shigella* sp. are markedly reduced at the AO‐EA extract's MIC when used to disrupt preformed biofilms. Specifically, only 15.15% ± 3.98% of *E. coli* (Figure 6G) and 14.9% ± 2.08% of *Shigella* sp. biofilm cells (Figure 6H) remained metabolically active at the MIC compared with the control. Collectively, these findings support the extract's antibiofilm activity, as evidenced by reductions in cell viability and metabolic activity.

Microscopy‐based analysis, particularly high‐resolution techniques, such as FE‐SEM, enables a detailed examination of bacterial cell shape, surface features, and biofilm structures. In this study, FE‐SEM was used to visually compare the biofilm structures formed by *E. coli* and *Shigella* sp. in the absence and presence of the extract. In the untreated control group, where bacteria were grown only in the culture medium without any extract, the micrographs showed a dense, continuous, and well‐organized biofilm. The cells appeared closely packed within an extracellular matrix, indicating robust attachment and maturation of the biofilm community (Figure 7A,C). Upon treatment with the extract at its MIC, a marked decline in biofilm formation was observed. The FE‐SEM images revealed a substantial reduction in the biofilm's overall biomass and coverage. Instead of a compact, layered structure, the treated samples displayed scattered, loosely associated cells, suggesting impaired adhesion and disruption of normal biofilm structure (Figure 7B,D). These morphological alterations indicate that the extract may compromise cell wall integrity and destabilize cell–cell and cell–surface interactions. Collectively, these FE‐SEM findings support the antibiofilm potential of the extract, as evidenced by both a reduction in biofilm density and visible physical damage to bacterial cells.

**Figure 7:**
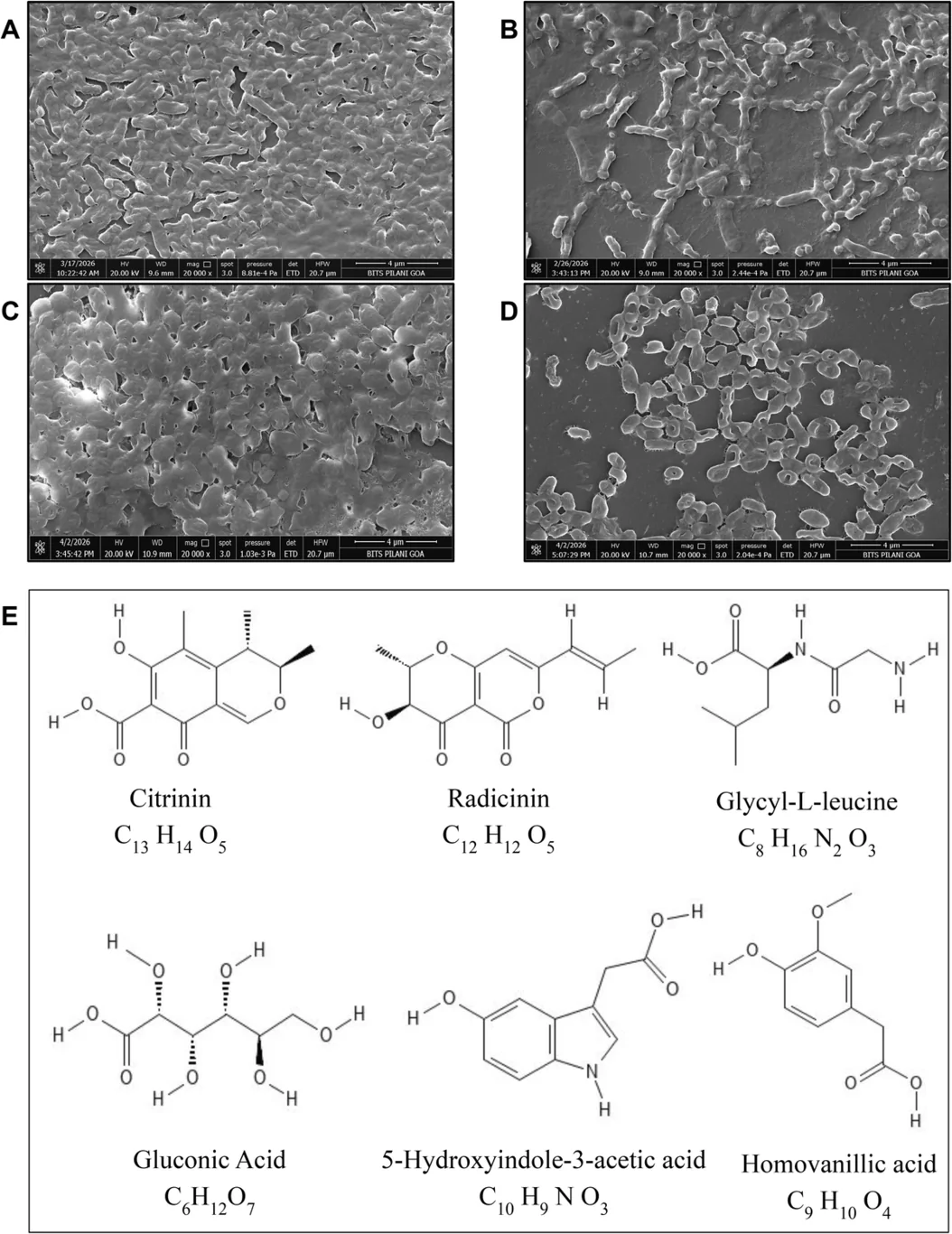
Scanning electron micrograph of biofilm formation and degradation assay. (A) 48 h untreated control of *Escherichia coli*, (B) 48 h *E. coli* biofilm treated with AO‐EA for 6 h, (C) 48 h untreated control of *Shigella* sp., and (D) 48 h *Shigella* sp. biofilm treated with AO‐EA for 6 h at ×20,000 magnification. (E) Structures of selected metabolites produced by various fungi and *Daldinia* sp., known for their potent antimicrobial properties. AO‐EA, ethyl acetate extract of *Daldinia* sp. from *Avicennia officinalis*.

### 3.6 LC‐HRMS Profiling Revealed the Presence of 15 Metabolites

The qualitative analysis of LC‐HRMS data for the ethyl acetate extract of the endophyte *Daldinia* sp. revealed various secondary metabolites, highlighting their diversity and distinct metabolic adaptations. This variation suggests that the endophyte has evolved specialized biochemical pathways that enable it to synthesize a range of bioactive compounds, thereby playing a crucial role in its ecological interactions within the mangrove ecosystem. The identified metabolites may contribute to the organism's ability to thrive in a challenging environment and also represent a valuable resource for the discovery of novel antimicrobial agents with therapeutic potential. Further investigations into the specific compounds present and their mechanisms of action could enhance our understanding of these adaptations and their applications in combating AMR. Metabolite analysis identified 15 distinct compounds using the mzCloud and ChemSpider databases. The identified metabolites are summarized in Table 3. The identified bioactive molecules were classified into several categories: antimicrobial peptides, carboxylic acids, alkaloids, polyketides, phenols, and other constituents. The extract contains several notable components, including citrinin, radicinin, homovanillic acid, azelaic acid, xanthine, 4‐acetamidobutanoic acid, and antimicrobial peptides, such as glycyl‐l‐leucine, 3‐[(4‐hydroxyphenyl)methyl]‐octahydropyrrolo[1,2‐*a*]pyrazine‐1,4‐dione, and 3‐(propan‐2‐yl)‐octahydropyrrolo[1,2‐*a*]pyrazine‐1,4‐dione. The chromatogram for the AO‐EA extract is shown in Figure S2. Furthermore, we have illustrated the structures of selected compounds known for their antimicrobial properties in Figure 7E using PubChem Sketcher V2.4.

**Table 3:** LC‐HRMS‐based metabolite profiling of AO‐EA extract.

S. no.	Name	Formula	Calculated MW (Da)	RT (min)	Annotated source: mzCloud search	Annotated source: ChemSpider search	Annotated DeltaMass (ppm)	Class	Reference
1	4‐Acetamidobutanoic acid	C_6_H_11_NO_3_	145.074	2.393	Full match	Partial match	0.72	Gamma amino acids	Xie et al. (2024)
2	Glycyl‐l‐leucine	C_8_H_16_N_2_O_3_	188.11612	4.765	Full match	Full match	0.15	Dipeptides	L. Spaggiari et al. (2026)
3	3‐[(4‐Hydroxyphenyl)methyl]‐octahydropyrrolo[1,2‐*a*]pyrazine‐1,4‐dione	C_14_H_16_N_2_O_3_	260.11628	7.593	Full match	Not the top hit	0.71	Cyclic peptides	W. Li et al. (2024) Nirwati et al. (2022)
4	3‐(Propan‐2‐yl)‐octahydropyrrolo[1,2‐*a*]pyrazine‐1,4‐dione	C_10_H_16_N_2_O_2_	196.12125	7.905	Full match	Partial match	0.36	Cyclic dipeptides	W. Li et al. (2024) Nirwati et al. (2022)
5	NP‐016455	C_11_H_18_N_2_O_4_	242.12675	8.329	Full match	Partial match	0.39	—	Khan and Mathur (2026)
									Nanda et al. (2024)
6	Anthranilic acid	C_7_H_7_NO_2_	137.04791	9.013	Full match	Partial match	1.71	Aminobenzoic acid	Teponno et al. (2017)
									C. S. Li et al. (2013)
7	NP‐011220	C_11_H_18_N_2_O_2_	210.13686	9.162	Full match	Partial match	0.14	—	Praptiwi et al. (2025)
8	5‐Hydroxyindole‐3‐acetic acid	C_10_H_9_NO_3_	191.05829	9.661	Full match	Full match	0.25	Carboxylic acids	Teuscher and Teuscher (1965)
									Huang et al. (2021)
9	Radicinin	C_12_H_12_O_5_	236.06841	10.07	Full match	Full match	−0.28	Aromatic ketones	Aldrich et al. (2015)
10	Citrinin	C_13_H_14_O_5_	250.08393	12.798	Full match	Full match	−0.77	Polyketides	B. Zhang et al. (2026)
11	Gluconic acid	C_6_H_12_O_7_	196.05768	1.206	Full match	Full match	−3.19	Carboxylic acids	Raj et al. (2024)
12	Citric acid	C_6_H_8_O_7_	192.02619	1.599	Full match	Full match	−4.21	Carboxylic acids	Sawant et al. (2018)
13	Azelaic acid	C_9_H_16_O_4_	188.10405	10.386	Full match	Full match	−4.31	Carboxylic acids	C. Spaggiari et al. (2023)
14	Homovanillic acid	C_9_H_10_O_4_	182.05704	10.761	Full match	Full match	−4.8	Phenols	M. Jia et al. (2025)
15	Xanthine	C_5_H_4_N_4_O_2_	152.03349	10.801	Full match	Full match	0.03	Alkaloids	B. Zhou et al. (2020)
									Kapri et al. (2022)

Abbreviations: AO‐EA, ethyl acetate extract of *Daldinia* sp. from *Avicennia officinalis*; LC‐HRMS, liquid chromatography with high‐resolution orbitrap mass spectrometry; MW, molecular weight; RT, retention time.

## 4 Discussion

Endophytic fungi are a highly diverse group that live within plant tissues for at least part of their life cycle, without causing any immediate or obvious effects on the host (Debbab et al. 2013). They promote plant growth and help plants tolerate biotic and abiotic stresses by producing a variety of secondary metabolites (Kaaniche et al. 2019). These metabolites give plants a competitive advantage over pathogens in natural environments and may also serve as promising candidates for new antibiotics against pathogenic bacteria when produced by endophytic fungi (Erb and Kliebenstein 2020). *Shigella* sp. are widespread and are the second leading cause of diarrheal deaths among people of all ages (Vaja et al. 2024). Diarrhea due to *E. coli* is also classified as a leading cause of mortality and morbidity in developing nations, associated with bacterial infections among other pathogens (Nasrollahian et al. 2024). The increasing resistance to broad‐spectrum antibiotics is complicating the treatment of many infections caused by these pathogens. Therefore, there is an urgent need for new therapeutic approaches to combat infections, which should be improved to enhance disease prevention and management (Vaja et al. 2024). Although fungi have been widely studied for bioactive compounds, those from unique niches, such as endophytic fungi, remain underexplored. They offer strong potential with diverse structures that can help address the urgent need for new, highly potent agents against emerging microbial pathogens and the growing threat of drug resistance (Fanele and Ndlovu 2023).

In this study, the fungal endophyte *Daldinia* sp. was isolated from the leaves of *A. officinalis*. These findings align with previous research on mangrove‐associated fungi. For example, the mangrove endophytic fungi *Alternaria longipes* (Ranganathan and Mahalingam 2018) and *Aspergillus flavus* (Ravindran et al. 2012) were isolated from the stems and leaves of *A. officinalis*. *Acremonium*, *Aspergillus*, *Penicillium*, *Curvularia*, *Cladosporium*, *Phoma*, and *Fusarium* have also been isolated from the leaves of *A. officinalis*, and the extracts from these fungi exhibited significant antimicrobial activity against a range of pathogenic bacteria and fungi, including *E. coli* (Job et al. 2015). Together, these studies emphasize the diversity and applications of mangrove‐associated fungal endophytes, highlighting their antimicrobial activity. Biological activity has been documented in the *Daldinia* genus. For example, Goswami et al. (2020) studied the antibacterial activity of *Daldinia concentrica* against both Gram‐positive and Gram‐negative bacteria, including *E. coli*, exhibiting an MIC of 780 μg/mL. Similarly, Ariantari et al. (2023) identified an endophytic fungus, *Daldinia eschscholtzii* AAB‐05, from *Antidesma bunius* leaves and tested its antimicrobial activity against bacterial pathogens, including *S. aureus*, *S. epidermidis*, and *Propionibacterium acnes*, with MIC values of 500, 1000, and 1000 μg/mL, respectively. Another study showed that the MIC of the crude extract of *D. eschscholtzii* against the tested bacteria, including *E. coli*, ranged from 0.312 to 5 mg/mL (Chutulo and Chalannavar 2020). The findings reported in several previous studies are consistent with our observations and support the view that different endophytic species belonging to the same genus can produce secondary metabolites with antimicrobial and antibiofilm activities.

In the present study, the AO‐EA extract exhibited antimicrobial activity, with inhibition zones ranging from 13.67 ± 0.57 to 17.33 ± 0.57 mm. Although this activity was lower than that generally observed with antibiotics, such as ciprofloxacin. The CLSI guidelines, described in Documents M02 (CLSI 2012) and M100 (CLSI 2015a), are intended for standardized antimicrobial susceptibility testing using pure, commercial antimicrobial agents and provide criteria for interpreting zone diameters and MIC values. In contrast, the AO‐EA extract is a crude mixture containing several compounds, some of which may be biologically active while others may have little or no antimicrobial activity. Therefore, the concentration of an individual active compound in the extract may be relatively low, and different compounds may act through distinct mechanisms. Ciprofloxacin, on the other hand, is a highly purified antimicrobial agent with a well‐established mechanism of action and is therefore expected to show greater activity than crude extract under standardized susceptibility testing conditions. The presence of inactive constituents in the extract may also affect the activity of the compounds responsible for the antimicrobial effect, resulting in an overall reduction in activity (Gonzalez‐Pastor et al. 2023). Further purification and enrichment of the active constituents may consequently improve the extract's antimicrobial activity.

The AO‐EA extract also showed pronounced inhibitory activity against the tested Gram‐negative pathogenic bacteria, as reflected by its MIC and MBC values. Similarly, another study reported that ethyl acetate crude extracts from *Rhizophora stylosa*, particularly from *Neopestalotiopsis protearum* HHL46, inhibited *P. adaceae*, *E. faecalis*, and methicillin‐resistant *S. aureus* (MRSA) with MIC values ranging from 0.0625 to 1 mg/mL. Extracts from *R. mucronata* also showed activity against human pathogens, particularly *Pestalotiopsis* sp. HQD6 and *N. protearum* HQD5 with MIC values ranging from 0.015 to 1 mg/mL (J. Zhou et al. 2022). Jouda et al. (2016) isolated three cytochalasins: 18‐methoxycytochalasin J, cytochalasin H, and cytochalasin J from the endophytic fungus *Phomopsis* sp. CAM240, obtained from *Garcinia kola* nuts. These compounds were active against *V. cholerae* NB2 and PC2, and *Shigella flexneri* SDINT, with MICs of 128–512 μg/mL. Together, these findings suggest that ethyl acetate extracts from endophytic fungi possess broad‐spectrum antimicrobial potential, supporting their further exploration as promising sources of novel anti‐infective agents.

Quick identification of drug effectiveness against bacterial pathogens is essential for detecting potentially resistant strains and facilitating more rational antimicrobial use. SEM can be utilized for visual detection as a rapid measure of the antimicrobial effect (Hisada et al. 2023). Nath and Joshi (2015) demonstrated that the crude ethanolic extract of the endophytic fungus *Glomerella acutata* EF15, isolated from *Hibiscus sabdariffa* L., exhibited strong antimicrobial activity against *K. pneumoniae* and *E. coli* and caused cells to appear sticky and shrunken at 4× MIC, indicating ultrastructural damage. A similar activity was observed in the ethyl acetate extract of *Muscodor* sp. IBRL OS‐94 from *Ocimum sanctum* against Gram‐negative bacteria, including *Yersinia enterocolitica, E. coli*, and *Shigella boydii*. SEM images of *Y. enterocolitica* cells treated with the extract showed marked structural damage, with cells appearing irregular, shrunken, and flaccid. Surface cleavages were also observed, indicating a disruption of bacterial cell integrity (Mat Jalil et al. 2020). A similar pattern was observed in our study during FE‐SEM analysis over a period of 3 h. Treatment at the MIC caused clear morphological changes in both *E. coli* and *Shigella* sp. The treated cells appeared deformed and wrinkled, with disrupted or broken structures and noticeable changes in their overall shape and size. Taken together, these observations suggest that extracts EF15, AO‐EA, and IBRL OS‐94 possess antibacterial activity and can cause substantial structural damage to Gram‐negative bacterial cells.

Flow cytometry has gained increasing importance in recent years for studying the effects of bioactive compounds on pathogenic microorganisms using fluorescent probes. The technique has also been considered a promising next‐generation approach for antimicrobial susceptibility testing (Duquenoy et al. 2020). Mycelial polysaccharides (PAT) obtained from *Armillariella tabescens* have been reported to exhibit strong antibacterial activity against *E. coli* (K. Zhang et al. 2022). In that study, SEM and CLSM observations showed that PAT affected the cell wall and membrane of *E. coli*, resulting in pronounced morphological damage and increased intracellular ROS levels. The flow cytometry results further indicated that PAT induced apoptosis in *E. coli*, with the apoptosis rate increasing at higher concentrations. The effects observed with AO‐EA in our study were comparable to those reported in these findings. AO‐EA caused damage to the cell wall and membrane of both *E. coli* and *Shigella*. At 2× MIC, 84.04% ± 5.89% of *E. coli* cells and 62.15% ± 1.77% of *Shigella* cells were PI‐positive, indicating disruption of membrane integrity. CLSM observations also showed a marked increase in ROS levels in AO‐EA‐treated cells compared with the untreated controls. These findings suggest that the antibacterial activity of AO‐EA is associated with disruption of membrane integrity and ROS‐mediated cell death.

The AO‐EA extract also showed substantial antibiofilm activity in the present study. It inhibited the formation of new biofilms by *E. coli* and *Shigella* by 93.03% ± 1.65% and 89.38% ± 0.91%, respectively. The extract was also effective against preformed biofilms, producing reductions of 86.20% ± 3.35% for *E. coli* and 83.02% ± 1.61% for *Shigella*. These results are significant because, based on the published criteria for interpreting antibiofilm activity, inhibition greater than 50% is considered good activity (Adeyemo et al. 2022). In this case, the AO‐EA extract not only exceeded this threshold but also demonstrated effectiveness comparable to the positive control, ciprofloxacin, with over 90% inhibition. It can be concluded that the AO‐EA extract exhibits strong antibiofilm activity, aligning with established standards and suggesting its potential as an effective antibacterial agent for biofilm‐related infections.

These inhibitory levels are comparable to those reported for metabolites from *Alternaria alternata* 13A, which reduced Gram‑negative biofilm formation by 40%–60% (Qader et al. 2021). An 80% inhibition is observed at 64 μg/mL by emodin, physcion, and related indole derivatives from *Eurotium chevalieri* KUFA0006 against *S. aureus* and *E. coli* (May Zin et al. 2017). In contrast, dihydroauroglaucin from *Aspergillus amstelodami* exhibited strong activity against established Gram‑negative biofilms, with low MIC values against *P. aeruginosa* (Fathallah et al. 2019). An endophytic fungus, *A. niger* AP5, was isolated from the leaves of *Phragmites australis*. The ethyl acetate extract of AP5 (AP5‐EtOAc) showed strong antibacterial activity against *S. aureus, E. coli*, and *Klebsiella* spp. Additionally, the AP5‐EtOAc extract exhibited significant antibiofilm activity, particularly against *S. aureus* and *P. aeruginosa* (Abdelgawad et al. 2022). Priya et al. (2025) investigated the antibiofilm potential of *Acrophialophora fusispora* DAOL8, an endophytic fungus isolated from *A. officinalis*, against four biofilm‐forming pathogens, including *E. coli*. Collectively, these results closely align with the findings of our study, which shows promising antibiofilm activity. The findings suggest that the AO‐EA extract is a promising candidate for use as a fungal‐derived antibiofilm agent. This extract demonstrates effective dual action against both the formation of new biofilms and the disruption of existing biofilms associated with Gram‐negative pathogens.

In our study, LC‐HRMS analysis of the ethyl acetate extract of *Daldinia* sp. identified several putative compounds that have not been previously reported from this genus. However, these metabolites have also been reported from other fungal sources, where they have shown antibacterial activity. Their presence in the AO‐EA extract may therefore be associated with the antibacterial effect observed in our study. Although the compounds isolated from *Daldinia* sp. are related to the metabolites identified in our LC‐HRMS analysis. The compounds are not exactly the same; they fall within the same chemical class, such as phenols and carboxylic acids. For instance, the two polyphenols dalesconosides A and C were previously identified from the *Ceriops tagal*‐derived fungus *Daldinia eschscholzii* MCZ‐18. These compounds have exhibited antibacterial activity against pathogenic organisms, including *E. coli* (Yu et al. 2024). Moreover, a study conducted by Mishra et al. (2020) demonstrated that 2,4‐di‐tert‐butylphenol, a member of the phenol class and isolated from the endophytic fungus *D. eschscholzii* found in the leaves of *Tridax procumbens*, significantly reduced biofilm formation by *P. aeruginosa*. 3,4,5‐Trimethoxybenzoic acid and fura carboxylic acid, both carboxylic acids, were isolated from *D. eschscholtzii* obtained from *Abutilon indicum* L. and exhibited antibacterial and antibiofilm activity (Musthafa et al. 2024).

Our research has revealed a range of bioactive metabolites produced by the endophyte *Daldinia* sp., highlighting significant potential in antibiotic development. Among these metabolites, citrinin and its derivatives, characterized by structures, such as benzopyran, quinone‐pyran, and benzofuran, have gained considerable attention. In recent years, several citrinin derivatives and dimers have been reported from fungal sources, with a wide range of biological activities (B. Zhang et al. 2026). Of particular relevance, citrinin isolated from the endophytic fungus *Penicillium rubens* EF 363, associated with the mangrove plant *Ceriops tagal*, was recently reported to show antimicrobial activity against several pathogens, including *E. coli* (Revathy et al. 2026). Similarly, radicinin obtained from the endophytic fungus *Cochliobolus* sp. associated with grapevine (*Vitis vinifera* L.) was found to exhibit antibacterial activity against *Xylella fastidiosa* (Aldrich et al. 2015). Antibacterial activity has also been reported for metabolites identified from other endophytic fungi. For example, the ethyl acetate extract of *Alternaria alstroemeriae*, isolated from *Fagopyrum dibotrys*, showed significant activity against *E. coli*. LC–MS analysis of this extract revealed several compounds, including 4‐acetamidobutanoic acid, azelaic acid, homovanillic acid, citric acid, and anthranilic acid, which may be responsible for the observed bioactivity (Xie et al. 2024). In addition, some alkaloid‐producing fungi, including *Claviceps purpurea*, produce 5‐hydroxyindole‐3‐acetic acid, which has been reported to possess antimicrobial activity against *Helicobacter pylori* (Teuscher and Teuscher 1965; Huang et al. 2021). Likewise, *A. niger* has been reported as a producer of gluconic acid, which has demonstrated antimicrobial activity against pathogens, such as *E. coli* and *Shigella* (Raj et al. 2024; Nieto‐Peñalver et al. 2014). Tea‐derived fungi, specifically *A. ustus* and *A. tamarii*, were found to produce xanthine and its derivatives, which were identified using HPLC. A review of the literature indicates that various xanthines and their derivatives have been identified as antimicrobial agents, demonstrating activity against pathogens, such as *E. coli* (B. Zhou et al. 2020; Kapri et al. 2022). The natural chemical compounds NP‐016455 (Khan and Mathur 2026) and NP‐011220 (Praptiwi et al. 2025) have been identified from fungal sources, *A. flavus* and *Fusarium equiseti*, respectively. Analysis by LC‐HRMS has confirmed the presence of these compounds in their respective extracts, which exhibit antimicrobial activity, indicating their potential as bioactive agents for pharmaceutical applications. In addition to these small molecules, antimicrobial peptides are gaining attention in pharmaceutical development due to their specificity and enhanced interactions. Recent findings have highlighted various endophytic peptides, suggesting that endophytes may be a valuable source of peptide‐based therapeutics. The current interest in antimicrobial membrane‐active peptides has made them subjects of extensive research worldwide (Abdalla and Matasyoh 2014). Our study identified dipeptides and cyclic peptides, including glycyl‐l‐leucine (L. Spaggiari et al. 2026), 3‐[(4‐hydroxyphenyl)methyl]‐octahydropyrrolo[1,2‐*a*]pyrazine‐1,4‐dione, and 3‐(propan‐2‐yl)‐octahydropyrrolo[1,2‐*a*]pyrazine‐1,4‐dione (W. Li et al. 2024; Nirwati et al. 2022). The evidence demonstrates that putative compounds identified by LC‐HRMS may significantly contribute to the antibacterial and antibiofilm properties of the AO‐EA extract.

Overall, these findings highlight the potential of mangrove‐derived endophytic fungi as invaluable sources of strong antimicrobial and antibiofilm compounds for promising therapeutic applications against infections caused by resistant bacteria.

## 5 Conclusion

Mangrove endophytic fungus *Daldinia* sp., isolated from leaves of *A. officinalis* in Goa, produced ethyl acetate extract (AO‐EA) with strong antibacterial activity. The extract showed clear zones of inhibition, measurable MIC and MBC values, and significant inhibition of bacterial growth. Microscopy and flow cytometry revealed cell membrane damage and increased cell death in *E. coli* and *Shigella* sp. At the same time, CLSM confirmed that the extract induces ROS generation in these bacteria, contributing to its antimicrobial effect. The AO‐EA extract from mangrove‐derived endophytic fungi showed strong antibiofilm and antimicrobial effects against *E. coli* and *Shigella* sp. It prevented about 90% of new biofilm formation, disrupted over 80% of existing biofilms, and greatly reduced the metabolic activity and viability of remaining cells, as confirmed by MTT assays and FE‐SEM imaging. LC‐HRMS profiling suggests the presence of various metabolites with antimicrobial properties. These results highlight the extract AO‐EA as a source of therapeutic antimicrobial and antibiofilm compounds.

## Author Contributions

**Nickita Dutta:** writing – original draft, methodology, data curation, investigation. **Nutan Kumari:** validation, visualization, data curation. **Anushka Lather:** software, validation, visualization, data curation. **Kundan Kumar:** conceptualization, funding acquisition, writing – review and editing, project administration, resources, supervision.

## Ethics Statement

The authors have nothing to report.

## Conflicts of Interest

The authors declare no conflicts of interest.

## Supporting information


Supporting File

## Data Availability

The data that support the findings of this study are included in this article and its Supporting Information Material. The data generated and/or analyzed during the current study are not publicly available and will be made available on request.
