# Supplementary material for: Antimicrobial and Antibiofilm Activity of an Endophytic Fungus, Daldinia sp. Isolated From Avicennia officinalis
Source: Microbiologyopen. 2026 Sep 29;15(5):e70422. doi: 10.1002/mbo3.70422 (PMC13622445; doi:10.1002/mbo3.70422)
Supplement: Supplementary file 1 [file MBO3-15-e70422-s001.docx]

**FIGURE 1** Antimicrobial activity (agar well diffusion assay) of the extract at 20 mg/mL concentration obtained from the endophyte *Daldinia* sp. against (A) *E. coli* and (B) *Shigella* sp. EA- Ethyl acetate extract. M- Methanolic extract

**FIGURE 2** Liquid chromatography-High resolution mass spectrometry (LC-HRMS) spectrum of AO-EA in both (+) and (-) ion modes.

**Table 1**. Antimicrobial activity (agar well diffusion assay) of the extract at 20 mg/mL concentration obtained from the endophyte *Daldinia* sp. against (A) *E. coli* and (B) *Shigella* sp. EA- Ethyl acetate extract. M- Methanolic extract

| **Pathogens** | **Zone of Inhibition (mm)**  **Conc. (mg/mL)** | |
| --- | --- | --- |
|  | **EA** | **M** |
| ***E. coli*** | 17.33 ± 0.57 | 12.0 ± 0.81 |
| ***Shigella* sp*.*** | 16.33 ± 0.57 | 11 ± 0.5 |

**Table 2** PI uptake percentage in *E. coli* and *Shigella* cells, comparing the effects observed before and after the introduction of the AO-EA extract and 70% ethanol.

| ***E. coli*** | **PI uptake (%)** | ***Shigella* sp.** | **PI uptake (%)** |
| --- | --- | --- | --- |
| **Unstained control** | 0 | **Unstained control** | 0 |
| **Stained control** | 12.72 ± 5.13 | **Stained control** | 13.92 ± 8.68 |
| **AO-EA (2x MIC)** | 84.04 ± 5.89 | **AO-EA (2x MIC)** | 62.15 ± 1.77 |
| **70% Ethanol** | 96.33 ± 0.97 | **70% Ethanol** | 99.35 ± 0.44 |
